# The relationships between pancreatic T2* values and pancreatic iron loading with cardiac dysfunctions,  hepatic and cardiac iron siderosis among Egyptian children and young adults with β-thalassaemia major and sickle cell disease: a cross-sectional study

**DOI:** 10.12688/f1000research.25943.2

**Published:** 2021-06-07

**Authors:** Khaled Salama, Amina Abdelsalam, Hadeel Seif Eldin, Eman Youness, Yasmeen Selim, Christine Salama, Gehad Hassanein, Mohamed Samir, Hanan Zekri

**Affiliations:** 1 Department of Pediatrics, Faculty of Medicine, Cairo University, Cairo, Egypt; 2 Department of Radiodiagnosis, Faculty of Medicine, Cairo University, Cairo, Egypt; 3 Department of Medical Biochemistry, National Research Centre, Giza, Egypt

**Keywords:** Iron overload, pancreatic iron, Thalassemia major, Sickle cell disease, Tissue Doppler Imaging

## Abstract

**Background:** Cardiac, hepatic and pancreatic T2* measured by magnetic resonance imaging (MRI) has been proven to be an accurate and non-invasive method for measuring iron overload in iron overload conditions. There is accumulating evidence that pancreatic iron can predict cardiac iron in young children because the pancreas loads earlier than the heart. The aim of our study was to assess the relationships between pancreatic T2* values and pancreatic iron loading with cardiac dysfunctions and liver and cardiac iron ****among patients with β-thalassaemia major (βTM) and sickle cell disease (SCD).

**Methods:** 40 βTM and 20 transfusion-dependant SCD patients were included along with 60 healthy age and sex-matched controls. Echocardiography and Tissue Doppler Imaging were performed for all subjects as well as the control group.  Hepatic, cardiac and pancreatic iron overload in cases were assessed by MRI T2*.

**Results:**  The mean age of our patients was 13.7 years with mean frequency of transfusion/year 12. Mean cardiac T2* was 32.9 ms and mean myocardial iron concentration was 0.7 mg/g; One patient had cardiac iron overload of moderate severity. Mean pancreatic T2* was 22.3 ms with 20 patients having mild pancreatic iron overload.

Pancreatic T2* correlated positively peak late diastolic velocity at septal mitral annulus (r=0.269, p=0.038), peak early diastolic velocity at tricuspid annulus (r=0.430, p=0.001) and mitral annular plane systolic excursion (r=0.326, p=0.01); and negatively with end systolic pulmonary artery pressure (r=-0.343, p=0.007) and main pulmonary artery diameter (MPA) (r=-0.259, p=0.046). We couldn’t test the predictability of pancreatic T2* in relation to cardiac T2* as only one patient had cardiac T2*<20 ms.

**Conclusion**: There was a relationship between pancreatic iron siderosis with cardiac dysfunction in multi-transfused patients with βTM and SCD. No direct relation between pancreatic iron and cardiac siderosis was detected.

## Introduction

The hallmark of chronic hemolytic anemias (CHAs) is premature destruction of erythrocytes ^1^. In Egypt, the common forms of inherited CHAs include β-thalassemia major (βTM) and sickle cell disease (SCD) ^2^. The main lines of therapy is blood transfusion and iron chelation. Quality of life and life expectancy have markedly improved since the introduction of oral iron chelators. However, death from cardiomyopathy and heart failure in these patients remains high, possibly because of the heavy transfusion burden ^3^ or the generation of reactive oxygen species induced by excess iron ^4^.

Consequently, it is important to detect silent cardiac dysfunction early before the development of symptomatic heart disease ^5^. Currently, several methods have been used in clinical studies for the determination of cardiac affection in iron overload conditions. Meanwhile, their uses are limited in βTM and SCD patients due to controversy on their reliability as well as their high cost. The aim of this study was to assess the relationships between pancreatic T2* values and pancreatic iron loading with cardiac dysfunctions and liver and cardiac iron in multi-transfused Egyptian patients with βTM and SCD.

## Methods

### Patients

40 βTM and 20 SCD patients aged ≥7 years with the onset of regular transfusions before the age of 2 years and receiving regular transfusions (≥ 3 transfusions per year with total volume ranging from 100 to 150 cc/kg/year according to Gale *et al.*, 2011) ^6^ following up at the hematology outpatient clinic, Cairo University Children’s Hospital, Cairo, Egypt during the study period (from June, 2017 to June, 2018) were enrolled in the study.

Patients with congenital or acquired heart diseases, hypertension, heart failure, cardiac drug usage, known risk factors for secondary pulmonary hypertension, acute febrile illness at enrollment or those with contraindications or inability to undergo magnetic resonance imaging (MRI) without sedation were excluded.

In addition, 60 healthy subjects with the same age and gender referred to our hospital for routine checkup were selected as the control group.

Demographics, transfusion history, and information concerning iron chelation and therapy were obtained by through patient interview during routine check-ups and chart review. Labs, including complete blood picture with blood indices, reticulocytic count and serum ferritin, were performed as routine labs done during check-ups.

The study was approved by the ethics and scientific research committee of Cairo University, Faculty of Medicine (ethical clearance number, I-060317) and the study was conducted in accordance with Cairo University’s laws for human research. Written informed consent was obtained from participants and parent/guardians in the case of children <18 years. Assent was obtained in addition from children <18 years.

### Cardiac T2*

All patients were scheduled for cardiac, hepatic and pancreatic MRI T2* at the Radiology Department, using a Philips Achiva, Netherland (1.5 Tesla) superconducting magnet with a Torso XL coil.

Scans were synchronized to the cardiac cycle using standard electrocardiogram gating. We then took a single 10 mm-thick short axis, mid ventricular slice positioned half way between the base and the apex of the left ventricle with repition time (TR) 20 ms and multiple echo times (TEs) (2.4, 4.6, 6.8, 9.1 and 11.3ms). Flip angle 30 and field of view (FOV) 320 mm. A fitting curve algorithm using a monoexponential decay with a constant offset (S=S0e-TE.R2*+C; S is the signal intensity, S0 is the signal intensity at TE=0 ms, and C is a constant) was applied to determine the T2* value. Results of cardiac T2* and myocardial iron concentration (MIC) measurements were considered as discontinuous variables and were classified as follows: normal iron concentration (T2* >20, MIC < 1.16 mg/g), light (T2* 15–20, MIC 1.16- 1.65 mg/g), moderate (T2* 10–15, MIC 1.65- 2.71 mg/g) and severe (T2* <10, MIC > 2.71 mg/g) ^7^.

### Liver and pancreas T2*

The liver T2* was measured by imaging a single trans-axial slice (10 mm) through the center of the liver. The pancreas T2* was measured by imaging a single trans-axial slice (10 mm) through the head of the pancreas with TR 20 ms and multiple TEs (2.4, 4.6, 6.8, 9.1 and 11.3ms) as well as through the body and mean region of interest was taken. Results of hepatic and pancreatic T2* measurements were considered as discontinuous variables and were classified as follows: normal iron concentration (T2* >11.4, liver iron concentration (LIC) < 2 mg/g), light (T2* 3.8–11.4, LIC 2.0- 7.0 mg/g), moderate (T2* 1.8–3.8, LIC 7.0–15.0 mg/g) and severe (T2* <1.8, LIC > 15 mg/g) ^8^.

We divided our patients into two groups according to LIC, normal <7 mg/g or overload >7 mg/g.

### Echocardiography examination

Trans-thoracic two dimensional (2D) guided (M mode) and doppler echocardiogram were performed with a Hewlett-Packard 5500 SONOS ultrasonic machine phased array sector scanner with the 4 and 8 MHZ probes according to age. M-mode, 2D and doppler echocardiographic parameters were averaged over three cardiac cycles and all echocardiographic measurements were performed according to the guidelines for performance of echocardiogram by American Society of Echocardiography ^9^. Tissue Doppler Imaging was performed for all patients and 60 – age and sex-matched – healthy subjects as a control group. Systolic function was assessed through measuring peak systolic (S’) myocardial velocities at both the septal and lateral mitral and free-wall tricuspid annulus. Diastolic function was assessed through measuring peak early diastolic (E’) and peak late diastolic (A’) myocardial velocities at both the septal and lateral mitral and free-wall tricuspid annulus. The ratio of E to E’ velocity (E/E’) was computed as a surrogate of LV filling pressure ^10^.

### Statistical analysis

The statistical package SPSS version 25 was used for data analysis. Mean, SD, and range were used for describing quantitative variables. The χ ^2^ test was used to compare qualitative variables between groups. The t-test was used to compare quantitative variables in parametric data. The Mann-Whitney test was used instead of the t-test for nonparametric data. Univariate correlations among the biological markers of iron metabolism and MRI LIC will be studied with Spearman’s rank-order correlation coefficient. Receiver-operator characteristic (ROC) curves will be used to analyze the capacity of serum ferritin to predict MRI-based hepatic iron overload, and to identify optimal test and threshold values. P-values <0.05 was considered significant and P values <0.01 were considered highly significant.

## Results

**Figure 1.**
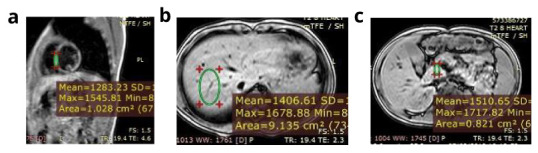
Magnetic resonance imaging of a patient with moderate cardiac iron overload, normal hepatic and pancreatic iron concentration. **a**) Calculated myocardial T2* equals 13.3 ms and myocardial iron concentration equals 1.91 mg/g (moderate cardiac iron overload). **b**) Calculated hepatic T2* equals 26.5 ms and liver iron concerntration equals 0.60 mg /g (no hepatic iron overload). **c**) Calculated pancreatic T2* equals 40 ms (no pancreatic iron overload). *Conclusion:* The patient has moderate cardiac iron overload, normal hepatic and pancreatic iron concentration.

In total, 40 βTM, 20 SCD and 60-age and sex matched subjects were included in the study. The study group consisted of 33 (55%) men and 27 (45%) women. Their mean age was 13.7 (±4.4) years and mean age at diagnosis was 12.4 (±8.6) months. A total of 40 (66.7%) patients were splenectomised (33 with βTM and 7 with SCD), and 58 patients (96.7%) were receiving a chelator (39 βTM; 19 SCD; p=0.6) for a duration ranging from 6 to 120 month (median 48 months). Assessment of the iron overload status of the studied patients revealed that 34 (56.7%) patients had average serum ferritin exceeding 2500 ng/ml, 10 (16.7%) had LIC ≥7 mg Fe/g dry liver weight (dw), 20 patients (33.3%) showed mild pancreatic iron overload and only one patient had evidence of cardiac iron overload of moderate severity ( Figure 1). Other demographic and laboratory data are illustrated in Table 1.

**Table 1.** Clinical, laboratory and iron profile data of the studied patients. Data are expressed as mean±SD (range).

Parameters	Study group (n=60)	β- thalassaemia major (n=40)	Sickle cell disease (n=20)	P-VALUE
Age (years)	13.7 ± 4.4 (7 – 24)	14.3 ± 4.5 (7 – 20)	12.6 ± 4.2 (7 – 24)	0.083
Male/Female	33/27	20/20	13/7	0.279
Weight (kg)	-1.1 ± 0.8 (-3 to 1)	-1.2 ± 0.8 (-3 – 0.7)	-1 ± 0.9 (-2.7 – 1)	0.409
Height (m)	-1.5 ± 1.5 (-6.3 – 5)	-1.5 ± 1.7 (-6.3 – 5)	-1.3 ± 1 (-3 – 0.8)	0.869
Hemoglobin (g/dl)	7.1 ± 1.4 (4.5 – 13.2)	6.9 ± 1.1 (4.5 – 9.3)	7.6 ± 1.8 (5.4 – 13.2)	0.246
Platelets (/mm ^3^)	568.8 ± 290.3 (143 – 1324)	650.3 ± 282.1 (203 – 1324)	405.8 ± 237.5 (143 – 978)	0.001 *
Serum ferritin (ng/ml)	3248.9 ± 2546.2 (200.9 – 13800)	3737.2 ± 2845.5 (719.2 – 13800)	2272.2 ± 1416.5 (200.9 – 6036)	0.050 *
Liver iron concentration (mg/g)	4.7 ± 2.6 (0.6 – 11.7)	4.8 ± 2.7 (0.6 – 11.7)	4.4 ± 2.4 (0.6 – 8.5)	0.906
Hepatic T2 *(ms)	8 ± 6.2 (2.3 – 26.5)	7.4 ± 5.1 (2.3 – 25)	9.2 ± 8 (3.1 – 26.5)	1.000
Cardiac T2 *(ms)	32.9 ± 12.4 (13.3 – 102.1)	32 ± 9.1 (20.3 – 60.6)	34.7 ± 17.5 (13.3 -102.1)	0.605
Myocardial iron concentration (mg/g)	0.7 ± 0.3 (0.1 – 1.9)	0.7 ± 0.2 (0.1 – 1.2)	0.7 ± 0.3 (0.2 – 1.9)	0.500
Pancreatic T2 * (ms)	22.3 ± 17.5 (4.5 – 116)	17.1 ± 11.7 (4.5 – 50.7)	32.6 ± 22.5 (5.7 – 116)	<0.0001 *

*Statistically significant, **ms=**milliseecond, SD=Standard deviation.

**Figure 2.**
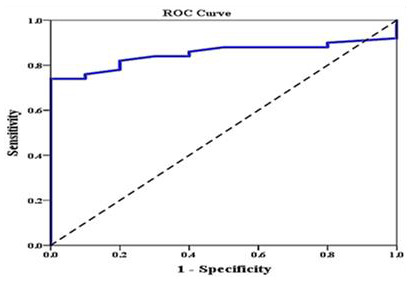
ROC curve analysis to explore the discriminant ability of pancreatic T2 in differentiating those with high liver iron concentration (LIC) (>7) from those with low LIC (<7).

Investigating the relation between different iron overload parameters revealed that serum ferritin correlated negatively with pancreatic T2* and positively with LIC. We divided our patients into two groups according to LIC, normal <7 or overload >7 (LIC is our gold standard), and this was plotted against pancreatic T2* using ROC curve. We found that area under the curve of pancreatic T2* was 0.851 (95% CI: 0.757–0.945). The sensitivity of pancreatic T2* in predicting LIC was 82% and specificity was 80% at a cutoff value ≥10.1 ( Figure 2). We failed to test the predictability pancreatic T2* in relation to cardiac T2* as nearly all patients have cardiac T2 > 20 and only. One patient has cardiac T2 < 20. ROC curve analysis couldn’t be done.

Echocardiographic assessments of the studied βTM and SCD patients were compared to the control group and the results are illustrated in Table 2. Mitral E/A ratio >2 was found in 18 (30%) patients indicating diastolic dysfunction of restrictive pattern. None of our patients had a mitral E/A ratio <1 denoting that none of them had diastolic dysfunction of impaired relaxation pattern. Statistically significant differences between patients and controls were detected in late diastolic myocardial velocities (A) and systolic myocardial velocities (S) at the basal mitral annulus of the lateral and septal walls being higher in patients than controls. Also peak systolic velocity at the tricuspid annulus of patients was significantly higher than controls ( Table 2).

**Table 2.** Echocardiographic and Tissue Doppler Imaging data of the studied patients and controls. Data are expressed as mean±SD (range).

Parameter	βTM (n=40)	SCD (n=20)	Control (n=60)	P-VALUE
FS	41.2 ± 5.8 (30 – 55)	40.1 ± 8.3 (30 – 55)	40.7 ± 3.4 (34 – 45)	0.779
EF	71.6 ± 6.5 (58 – 83)	69.7 ± 8.9 (58 – 83)	70.8 ± 5.8 (58 – 79)	0.792
TAPSE (mm)	28.6 ± 6.1 (9 – 37)	30.8 ± 3.8 (25 – 37)	22.1 ± 2.8 (14 – 27)	<0.0001 *
MAPSE (mm)	18.1 ± 2.7 (15 – 25)	16.7 ± 2.7 (15 – 25)	16.2 ± 1.9 (12 – 20)	0.019 *
ESPAP (mmHg)	32.1 ± 7.7 (20 – 44)	28.4 ± 9 (18 – 44)	21.3 ± 3.9 (17 – 32)	<0.0001 *
Mitral E/E'	6.9 ± 1.93 (3.5 – 10.8)	5.97 ± 2.49 (3.5 – 11)	6.83 ± 1.47 (4 – 11)	0.060
Mitral E/A	1.9 ± 0.6 (1.1 – 2.9)	1.7 ± 0.5 (1.1 – 2.8)	1.3 ± 0.3 (0.8 – 2)	<0.0001 *
Mitral annular velocity (septal annulus)				
E’(m/sec)	0.15 ± 0.03 (0.08 – 0.19)	0.15 ± 0.01 (0.11 – 0.17)	0.15 ± 0.03 (0.11 – 0.19)	0.457
A’(m/sec)	0.08 ± 0.04 (0.03 – 0.18)	0.1 ± 0.04 (0.06 – 0.18)	0.07 ± 0.01 (0.05 – 0.08)	0.031 *
S’(m/sec)	0.08 ± 0.02 (0.04-0.13)	0.09 ± 0.02 (0.06 – 0.13)	0.07 ± 0.01 (0.06 – 0.09)	0.004 *
Mitral annular velocity (lateral annulus)				
E’(m/sec)	0.17 ± 0.04 (0.11 – 0.28)	0.19 ± 0.04 (0.15 – 0.28)	0.16 ± 0.01 (0.13 – 0.18)	0.068
A’(m/sec)	0.1 ± 0.06 (0.04 – 0.22)	0.11 ± 0.05 (0.06 – 0.22)	0.06 ± 0.01 (0.05 – 0.1)	<0.0001 *
S’(m/sec)	0.09 ± 0.02 (0.05 – 0.14)	0.1 ± 0.02 (0.06 – 0.13)	0.08 ± 0.01 (0.07 – 0.09)	0.005 *
Tricuspid annular velocity				
E’(m/sec)	0.16 ± 0.06 (0.06 – 0.28)	0.18 ± 0.05 (0.12 – 0.28)	0.18 ± 0.02 (0.15 – 0.2)	0.183
A’(m/sec)	0.12 ± 0.05 (0.03 – 0.19)	0.12 ± 0.05 (0.06 – 0.19)	0.12 ± 0.01 (0.1 – 0.14)	0.986
S’(m/sec)	0.13 ± 0.04 (0.04 – 0.21)	0.14 ± 0.04 (0.08 – 0.21)	0.12 ± 0.01 (0.1 – 0.16)	0.006 *

*Statistically significant, **ms=**milliseecond, **βTM=**Beta thalassemia major **, SCD=**Sickle cell disease **, LIC=**Liver iron concentration **, MIC=**Myocardial iron concentration, SD=Standard deviation. **FS=**Fractional shortening **, EF=**Ejection fraction **, Mitral E/A=**Ratio of the peak velocity of early diastolic transmitral flow to the peak velocity of the late diastolic transmitral flow **, TAPSE=**Tricuspid annular plain systolic excursion **, MAPSE=**Mitral annular plane systolic excursion **, ESPAP=**End systolic pulmonary artery pressure, **Mitral E/ E'**= Ratio of the peak velocity of early diastolic transmitral flow to early diastolic mitral annular velocity **, Mitral E/A=**Ratio of the peak velocity of early diastolic transmitral flow to the peak velocity of the late diastolic transmitral flow **S'**=Peak systolic velocity, **E'**=Peak early diastolic velocity, **A'**=Peak late diastolic velocity.

### Pancreatic iron and cardiac dimensions and functions

We divided our patients according to grade of pancreatic iron; group (n=40) with normal pancreatic iron and group (n=20) with pancreatic iron overload. The pancreatic iron overload group showed higher LIC, higher end systolic pulmonary artery pressure (ESPAP) and lower tricuspid annulus peak early diastolic velocity compared to the group with normal pancreatic iron ( Table 3).

**Table 3.** Comparison of hepatic and cardiac iron and cardiac functions in relation to pancreatic iron.

Mean ± SD (range)	Normal (T2* >11.4) (n=40)	Mild (T2* 3.8- 11.4 ms) (n=20)	P value
LIC	4.06 ± 2.03 (0.6 – 7.13)	5.98 ± 3.17 (0.9 – 11.7)	0.032 *
MIC	0.67 ± 0.26 (0.16 – 1.9)	0.72 ± 0.24 (0.13 – 1.12)	0.140
FS	41.35 ± 7.51 (30-55)	39.9 ± 4.67 (30-44)	0.535
EF	71.2 ± 8.08 (58-83)	70.35 ± 5.76 (58-75)	0.478
ESPAP	29.63 ± 8.79 (18-44)	33.4 ± 6.64 (24-44)	0.046 *
E' (Lateral mitral annulus)	0.17 ± 0.04 (0.11 – 0.28)	0.18 ± 0.04 (0.12 – 0.28)	0.713
S' (Lateral mitral annulus)	0.1 ± 0.02 (0.05 – 0.14)	0.09 ± 0.02 (0.05 – 0.11)	0.147
A' (Lateral mitral annulus)	0.11 ± 0.06 (0.04 – 0.22)	0.09 ± 0.06 (0.04 – 0.22)	0.285
E' (Septal mitral annulus)	0.15 ± 0.02 (0.08 – 0.17)	0.16 ± 0.03 (0.08 – 0.19)	0.103
S' (Septal mitral annulus)	0.09 ± 0.02 (0.04 – 0.13)	0.08 ± 0.02 (0.04 – 0.12)	0.816
A' (Septal mitral annulus)	0.09 ± 0.05 (0.03 – 0.18)	0.07 ± 0.02 (0.03 – 0.11)	0.425
E' (Tricuspid annulus)	0.18 ± 0.06 (0.06 – 0.28)	0.14 ± 0.04 (0.06 – 0.21)	0.020 *
S' (Tricuspid annulus)	0.14 ± 0.05 (0.04 – 0.21)	0.13 ± 0.03 (0.04 – 0.16)	0.400
A' (Tricuspid annulus)	0.12 ± 0.05 (0.03 – 0.19)	0.12 ± 0.05 (0.03 – 0.19)	0.994

*Statistically significant, **LIC=**Liver iron concentration **, MIC=**Myocardial iron concentration, **FS=**Fractional shortening **, EF=**Ejection fraction, **ESPAP=**End systolic pulmonary artery pressure, **S'**=Peak systolic velocity, **E'**=Peak early diastolic velocity, **A'**=Peak late diastolic velocity.

Pancreatic T2* correlated positively with peak late diastolic velocity at septal mitral annulus, peak early diastolic velocity at tricuspid annulus and mitral annular plane systolic excursion (MAPSE). It correlated negatively with main pulmonary artery diameter (MPA) and ESPAP ( Table 4).

**Table 4.** Correlations of pancreatic T2*, liver iron concentration and ferritin with echocardiographic and tissue doppler variables.

	Pancreatic T2*		LIC		Serum ferritin	
	R	P	R	P	R	P
LVEDD (mm)	0.195	0.136	-0.130	0.322	-0.053	0.690
LVESD (mm)	-0.082	0.534	0.113	0.391	-0.029	0.826
FS	0.201	0.123	-0.135	0.305	0.078	0.554
EF	0.243	0.062	-0.152	0.245	0.075	0.569
RVDd (mm)	0.160	0.222	-0.268	0.038	-0.208	0.112
LA/Ao	-0.049	0.710	0.088	0.505	0.008	0.953
MPA (mm)	-0.259	0.046	-0.102	0.438	-0.056	0.669
ESPAP	-0.343	0.007	0.168	0.200	0.182	0.163
E' (Lateral mitral annulus)	0.054	0.680	-0.217	0.095	-0.038	0.772
S' (Lateral mitral annulus)	0.224	0.085	-0.313	0.015	-0.149	0.257
A' (Lateral mitral annulus)	0.167	0.203	-0.168	0.198	-0.066	0.617
E' (Septal mitral annulus)	-0.197	0.131	-0.007	0.959	0.128	0.328
S' (Septal)	0.008	0.953	-0.299	0.020	-0.053	0.688
A' (Septal)	0.269	0.038	-0.423	0.001	-0.201	0.124
E' (Tricuspid annulus)	0.430	0.001	-0.335	0.009	-0.184	0.158
S' (Tricuspid annulus)	0.203	0.120	-0.229	0.079	-0.023	0.862
A' (Tricuspid annulus)	0.012	0.928	-0.087	0.509	0.061	0.643
TAPSE	0.236	0.069	-0.082	0.536	-0.042	0.748
MAPSE	0.326	0.011	0.049	0.712	0.106	0.420
Mitral E/A	-0.172	0.189	0.189	0.149	0.142	0.278

**LVEDD=**Left ventricular end diastolic diameter **, LVESD=**Left ventricular end systolic diameter **, FS=**Fractional shortening **, EF=** Ejection fraction, **RVDd=**Right ventricular dimension at end diastole, **LA/Ao=**Ratio of the left atrial dimension to the aortic annulus dimension **, MPA=**Main pulmonary artery **, ESPAP=**End systolic pulmonary artery pressure, **S'**=Peak systolic velocity, **E'**=Peak early diastolic velocity, **A'**=Peak late diastolic velocity **, TAPSE=** Tricuspid annular plain systolic excursion **, MAPSE=**Mitral annular plane systolic excursion **, Mitral E/A=**Ratio of the peak velocity of early diastolic transmitral flow to the peak velocity of the late diastolic transmitral flow, **R=** Spearman correlation coefficient.

There was a significant negative correlation between LIC and right ventricular dimension at end diastole (RVDd) as well as the following Tissue Doppler parameters: peak systolic velocity at lateral and septal mitral annulus, peak late diastolic velocity at septal mitral annulus and peak early diastolic velocity at tricuspid annulus ( Table 4).

## Discussion

There is accumulating evidence that pancreatic iron can predict cardiac iron in young children ^11^. Because the pancreas takes up non-transferrin bound iron, generating reactive oxygen species of the heart, but earlier, it can be used as an early and strong marker of prospective risk of cardiac siderosis, with a 100% negative predictive value for cardiac iron accumulation ^12^. Unfortunately, we failed to test the predictability of pancreatic T2* in relation to cardiac T2* in our study as nearly all of our patients had normal cardiac T2* and only one patient was cardiac iron loaded. In spite of the extensive involvement of the liver and pancreas among our patients, almost absence of cardiac iron loading (n=1) was striking. Several factors affect the transport, storage and removal of iron in the different organs in iron overload conditions leading to the heterogenous distribution of storage iron in each organ measured by MRI T2* ^13^. Genetic background of thalassemic patients in Egypt might be implicated for the low prevalence of cardiac iron loading in our population in spite of very high serum ferritin and high LIC ^14^. In another study in Alexandria University performed on thalassemic patients, only 8.7% of cases were cardiac iron loaded ^15^. Similarly in SCD patients, Elalfy *et al.* found that cardiac siderosis was absent in transfusion dependant SCD patients with minimal or no pancreatic and moderate to severe hepatic iron loading ^16^.

Our results disagree with a study carried out on 131 βTM patients that reported increased cardiac iron in almost one third of patients and increased pancreatic iron in more than 70% of patients with good correlation between pancreatic and cardiac iron (r2= 0.52), with the pancreas loading a decade earlier than the heart ^11^. This difference might be explained by the smaller sample size, the design of our study, as well as the younger age group of our patients.

Despite the absence of cardiac iron deposition, there was significant difference between cases and controls (p<0.0001) in diastolic indices of LV (Trans-mitral E/A ratio) and mitral E/A ratio >2 was found in 30% indicating diastolic dysfunction of restrictive pattern. None of our patients had diastolic dysfunction of impaired relaxation pattern.

The diagnosis of subclinical LV systolic dysfunction can be detected by assessing MAPSE. MAPSE was significantly reduced in patients with chronic heart failure, with a good correlation between MAPSE and EF ^17– 19^. Among our patients the mean MAPSE was comparable between SCD and controls and was higher in βTM patients (p=0.019) when compared to SCD and controls. This indicates an absence of subclinical LV systolic dysfunction in our studied patients.

Up to one third of our patients had ESPAP ≥35 mmHg. Moreover, both βTM and SCD groups had significantly higher ESPAP than controls (p<0.0001). At the time of enrollment 66.7% patients had been splenectomized, also βTM patients had a higher platelet count; both factors could contribute to the higher ESPAP in thalassemia patients. This was in agreement with previous studies that reported splenectomy as one of the main risk factors of cardiac disease in such a group of patients ^20, 21^.

Comparing the myocardial velocity measurements of cases with controls; there were significant differences in late diastolic (A’) and systolic myocardial velocities (S’) at the basal mitral annulus of the lateral and septal walls (p<0.0001, p=0.005, p=0.031 and p=0.004 respectively) being higher in patients than controls. Also peak systolic velocity (S’) at the tricuspid annulus of patients was significantly higher than controls. These findings may be due to the chronic anemia and high cardiac output, which dominate the picture in children unlike other studies that reported that tissue Doppler systolic velocity was significantly lower in the βTM group compared to controls ^22^.

Minimal preliminary data have postulated a correlation between pancreatic iron overload and cardiac function in βTM and SCD patients. Our data revealed that pancreatic T2* correlated positively with peak late diastolic velocity at septal mitral annulus (r=0.269, p=0.038) and peak early diastolic velocity at tricuspid annulus (r=0.430, p=0.001); which means that pancreatic iron overloaded patients had some degree of left and right ventricular diastolic dysfunction respectively, though not yet cardiac loaded. Also patients with pancreatic iron overload had a higher ESPAP compared to those with normal pancreatic T2* (p=0.046), pancreatic T2* showed a weak negative correlation with ESPAP (r=-0.343, p=0.007) and with main pulmonary artery diameter (MPA) (r=-0.259, p=0.046). This means that pancreatic iron load might serve as a good indicator of the risk of developing pulmonary hypertension in βTM and SCD patients even before frank cardiac iron loading and before appearance of cardiac symptoms as well. Searching the literature, similar correlations between pancreatic T2* and the development of pulmonary hypertension and diastolic dysfunction could not yet be found. But Pepe *et al.*, in their study on a large cohort of βTM patients, reported that pancreatic iron was correlated to the LV EF, but not to the RV EF ^23^.

## Conclusion

Multi-transfused SCD and βTM patients with iron overload showed more iron deposition in the liver, followed by the pancreas with relative sparing of the heart. Assessment of pancreatic T2* in multi-transfused children and young adults with SCD and βTM can predict myocardial dysfunction detected by Tissue Doppler Imaging in the absence of abnormal indices of global ventricular dysfunction or even the appearance of symptoms and signs of overt heart failure. In fact, we recommend that pancreatic T2* should be considered as an additional tool to LIC and cardiac T2 * for evaluation and monitoring of young children with βTM and SCD at risk of iron overload. We also recommend that Tissue Doppler echocardiography should be applied in cardiac assessment among patients with chronic hemolytic anemia and should be performed at regular intervals.

## Data availability

### Underlying data

Open Science Framework: Iron overload parameters and early detection of cardiac disease among Egyptian children and young adults with β-thalassaemia major and sickle cell disease: a cross-sectional study, https://doi.org/10.17605/OSF.IO/58Q3D ^24^.

Data are available under the terms of the Creative Commons Zero "No rights reserved" data waiver (CC0 1.0 Public domain dedication).
