# Supplementary material for: The relationships between pancreatic T2* values and pancreatic iron loading with cardiac dysfunctions,  hepatic and cardiac iron siderosis among Egyptian children and young adults with β-thalassaemia major and sickle cell disease: a cross-sectional study
Source: F1000Res. 2021 Jun 7;9:1108. Originally published 2020 Sep 9. [Version 2] doi: 10.12688/f1000research.25943.2 (PMC8261758; doi:10.12688/f1000research.25943.2)
Supplement: Peer review history

# Peer review: Reviewer response for version 2

**DOI:** 10.5256/f1000research.56428.r86937

**Published:** 2021-07-06

**Authors:** Hadi Darvishi-Khezri

**Affiliations:** 1 Thalassemia Research Center (TRC), Hemoglobinopathy Institute, Mazandaran University of Medical Sciences, Sari, Iran

I have checked the authors' answers and their revisions on the manuscript. The changes and revisions are acceptable in my view.

Is the work clearly and accurately presented and does it cite the current literature?

Partly

If applicable, is the statistical analysis and its interpretation appropriate?

Partly

Are all the source data underlying the results available to ensure full reproducibility?

Yes

Is the study design appropriate and is the work technically sound?

Yes

Are the conclusions drawn adequately supported by the results?

Partly

Are sufficient details of methods and analysis provided to allow replication by others?

Yes

Reviewer Expertise:

NA

I confirm that I have read this submission and believe that I have an appropriate level of expertise to confirm that it is of an acceptable scientific standard.

# Peer review: Reviewer response for version 1

**DOI:** 10.5256/f1000research.28631.r72131

**Published:** 2020-10-20

**Authors:** Hadi Darvishi-Khezri

**Affiliations:** 1 Thalassemia Research Center (TRC), Hemoglobinopathy Institute, Mazandaran University of Medical Sciences, Sari, Iran

The article has been reviewed. The study meticulously investigated pancreatic, cardiac and liver iron loading, as well as cardiac dysfunctions among transfusion-dependent β-thalassaemia and sickle cell patients. The researchers tried to reveal the correlation between pancreatic iron loading with cardiac and liver iron siderosis and also cardiac dysfunction.

 Although the results of study are intriguing, several flaws still remain in the paper.

**Title**

- The title of article is not completely matched with the aim of study.
- The term of cardiac disease used in the title is rather wide. It would be better that more specific term would be chosen for the title of study. 

**Abstract**

- The first sentence of the result and method in the Abstract is similar, and a repetition as well (40 βTM and 20 transfusion-dependent SCD patients).
- The last sentence in the Abstract part reflects the results of the relationship between pancreatic T2* values and cardiac iron loading, which is not present in the aim of the study.
- The results of cardiac function were not reflected as part of results in the Abstract.
- It would be better the correlation coefficients (r) in the Abstract would be reported.
- The study did not assess the prediction of cardiac dysfunctions by pancreatic T2* values. So, a more appropriate substitutional sentence for the conclusion is “There was a relationship between pancreatic iron siderosis with cardiac dysfunction in these patients”.

**Introduction**

- The authors stated that the aim of the study is to investigate 1. Cardiac function 2. Cardiac iron loading 3. Their relation to pancreatic iron loading in patients with β-thalassemia major and sickle cell disease (mentioned in the Abstract, Introduction and Discussion sections). However, it seems that the study was established to assess the relationships between pancreatic T2* values and pancreatic iron loading with cardiac dysfunctions and liver and cardiac iron siderosis, not a prediction analyses.
- The last sentence in the first paragraph of the Introduction section is vague.

**Method **

- The logic for calculating sample size is missed in this study. The calculation of sample size or a power analysis is necessary.
- Whether the healthy controls has been matched based on age and sex or only age? The first sentence of the Results section in the Abstract part and the main text of the article is different.
- Usually serum ferritin levels of greater than 2000 ng/mL is considered as iron overload in transfusion dependent thalassemia [1]. Hyperferritinemia in non-transfusion dependent thalassemia is a serum ferritin higher than 800 ng/mL [2].

**Results**

- The number (%) of myocardial dysfunctions (systolic and diastolic), pancreatic, cardiac and liver iron siderosis were not clearly reported in the article.
- The authors could have used odds ratio to show the relationship between pancreatic T2* values with cardiac dysfunction, as well as the strength of the relationship.
- The categorization of patients based on LIC should have been placed in the Method section.
- In the second paragraph, the results related to the prediction of liver siderosis by pancreatic T2* values is not reflected in the aim of study.
- Figure 2 is not so meaningful.
- Based on the title of Table 3, LIC reported in Table 3 does not belongs to cardiac function.
- I expected to see the status of cardiac and liver MRIs according to pancreatic iron siderosis.
- The majority of correlations between pancreatic T2*, LIC and serum ferritin with cardiac indices measured by trans thoracic and tissue Doppler echocardiography were weak (correlation coefficient < 0.4). The correlation of LIC and peak late diastolic (A') was detected as a moderate strength of relationship (r = 0.423). The intensity of correlation between pancreatic T2* with E' was also moderate (r = 0.43).

 
**Discussion**

- The aim of the study should have been mentioned at the end of the Introduction part instead of at the beginning of the Discussion section.
- There is a disparate concept in the paper about the time of pancreatic and cardiac iron loading. In the Abstract it is written: “There is accumulating evidence that pancreatic iron can predict cardiac iron in young children because the pancreas loads earlier than the heart”. Conversely, in the second paragraph of the Discussion part it is written: “Cardiac iron overload occurs earlier than pancreatic iron overload, therefore pancreatic iron can predict cardiac iron in young children”.
- Wording and writing in the Discussion part is similar to the Results section. To some extent, the Discussion part is the repetition of the results section. Discussion should be the interpretation of the results and a comparison with other studies.
- There have been some limitations in this study, which is absent in the paper. The population of the study is heterogeneous, comprising with β-thalassemia major and sickle cell disease. As you can see, the difference of pancreatic T2* values between two groups (βTM and SCD patients) shown in Table 1 is significant. In addition, I expected to see several recommendations to make a contribution to set up future studies. 

Is the work clearly and accurately presented and does it cite the current literature?

Partly

If applicable, is the statistical analysis and its interpretation appropriate?

Partly

Are all the source data underlying the results available to ensure full reproducibility?

Yes

Is the study design appropriate and is the work technically sound?

Yes

Are the conclusions drawn adequately supported by the results?

Partly

Are sufficient details of methods and analysis provided to allow replication by others?

Yes

Reviewer Expertise:

Thalassemia, Clinical Science, Critical Care

I confirm that I have read this submission and believe that I have an appropriate level of expertise to confirm that it is of an acceptable scientific standard, however I have significant reservations, as outlined above.

## References

[1] : A prospective analysis for prevalence of complications in Thai nontransfusion-dependent Hb E/β-thalassemia and α-thalassemia (Hb H disease). *Am J Hematol* .93(5) : 10.1002/ajh.25046 623-629 10.1002/ajh.25046 29359464

[2] : Multiparametric Cardiac Magnetic Resonance Survey in Children With Thalassemia Major: A Multicenter Study. *Circ Cardiovasc Imaging* .2015;8(8) : 10.1161/CIRCIMAGING.115.003230 e003230 10.1161/CIRCIMAGING.115.003230 26253625

# Response

**Published:** 2021-04-23

**Authors:** yasmeen selim

**Affiliations:** Cairo University, Egypt

***Title***

**The title of article is not completely matched with the aim of study**

 You are right dear sir/I am going to modify the title

- **The term of cardiac disease used in the title is rather wide. It would be better that more specific term would be chosen for the title of study**
  I am going to change the term cardiac disease to a more specific term
***Abstract***
- **The first sentence of the result and method in the Abstract is similar, and a repetition as well (40 βTM and 20 transfusion-dependent SCD patients)**
  I am going to change this sentence
- The last sentence in the Abstract part reflects the results of the relationship between pancreatic T2* values and cardiac iron loading, which is not present in the aim of the study 
  The aim of our study was to investigate cardiac function and cardiac iron and their relation to pancreatic iron among patients with β-thalassaemia major (βTM) and sickle cell disease (SCD), So the last sentence in the Abstract part that reflects the results of the relationship between pancreatic T2* values and cardiac iron loading is actually mentioned in the aim of the study
- **The results of cardiac function were not reflected as part of results in the Abstract**
  The results of cardiac function were not reflected as part of results in the Abstract as we were restricted by the number of word count of the abstract so we only included the positive correlations with pancreatic T2 
- **It would be better the correlation coefficients (r) in the Abstract would be reported**
  I will add the r value
- The study did not assess the prediction of cardiac dysfunctions by pancreatic T2* values. So, a more appropriate substitutional sentence for the conclusion is “There was a relationship between pancreatic iron siderosis with cardiac dysfunction in these patients”
  I will change this sentence in the conclusion
***Introduction***
- The authors stated that the aim of the study is to investigate 1. Cardiac function 2. Cardiac iron loading 3. Their relation to pancreatic iron loading in patients with β-thalassemia major and sickle cell disease (mentioned in the Abstract, Introduction and Discussion sections). However, it seems that the study was established to assess the relationships between pancreatic T2* values and pancreatic iron loading with cardiac dysfunctions and liver and cardiac iron siderosis, not a prediction analyses.
  I am going to edit this part
- **The last sentence in the first paragraph of the Introduction section is vague**
  I am going to replace the last sentence in the first paragraph of the Introduction by a more understandable one
***Methods***
- **Whether the healthy controls has been matched based on age and sex or only age? The first sentence of the Results section in the Abstract part and the main text of the article is different**
  Healthy controls were both age and sex matched, It is just a typing error I will fix it
- **Usually serum ferritin levels of greater than 2000 ng/mL is considered as iron overload in transfusion dependent thalassemia [1]. Hyperferritinemia in non-transfusion dependent thalassemia is a serum ferritin higher than 800 ng/mL [2]**
  The estimation of serum ferritin levels is the most commonly employed test to evaluate iron overload in Beta Thalassemia Major. A target ferritin of approximately 1000 ng/ml is generally recommended standard practice in thalassaemia major (TIF Guidelines, 2000) and other forms of iron overload resulting from blood transfusion. When the
  serum ferritin level reaches 1000 ng/ml (usually after 10 ^th^ to 12 ^th^ transfusion), it is generally taken as the point to initiate iron chelation therapy.
***Results***
- **The number (%) of myocardial dysfunctions (systolic and diastolic), pancreatic, cardiac and liver iron siderosis were not clearly reported in the article**
  The Hepatic, Cardiac and pancreatic iron loading data are illustrate din table 1 and myocardial dysfunction data are illustrated in the third paragraph in results and table 2
- **The categorization of patients based on LIC should have been placed in the Method section**
  Categorization of patients based on LIC will be added in methods section
- In the second paragraph, the results related to the prediction of liver siderosis by pancreatic T2* values is not reflected in the aim of study
  The results related to the prediction of liver siderosis by pancreatic T2* values is not reflected in the aim of study as this was a secondary outcome not our primary one
- **Figure 2 is not so meaningful**
  Figure 2 is based on the secondary outcome that we found
- **Based on the title of Table 3, LIC reported in Table 3 does not belongs to cardiac function**
  I will change the title of table 3 
- The majority of correlations between pancreatic T2*, LIC and serum ferritin with cardiac indices measured by trans thoracic and tissue Doppler echocardiography were weak (correlation coefficient < 0.4). The correlation of LIC and peak late diastolic (A') was detected as a moderate strength of relationship (r = 0.423). The intensity of correlation between pancreatic T2* with E' was also moderate (r = 0.43)
  Though the correlations are weak but there still exist a correlation and this is actually what we found
***Discussion***
- **The aim of the study should have been mentioned at the end of the Introduction part instead of at the beginning of the Discussion section**
  I will remove this part
- **There is a disparate concept in the paper about the time of pancreatic and cardiac iron loading. In the Abstract it is written: “There is accumulating evidence that pancreatic iron can predict cardiac iron in young children because the pancreas loads earlier than the heart”. Conversely, in the second paragraph of the Discussion part it is written: “Cardiac iron overload occurs earlier than pancreatic iron overload, therefore pancreatic iron can predict cardiac iron in young children”**
  I am going to edit this part regarding Cardiac iron overload occurs earlier than pancreatic iron overload, therefore pancreatic iron can predict cardiac iron in young
  children as it is really contradictory
- There have been some limitations in this study, which is absent in the paper. The population of the study is heterogeneous, comprising with β-thalassemia major and sickle cell disease. As you can see, the difference of pancreatic T2* values between two groups (βTM and SCD patients) shown in Table 1 is significant. In addition, I expected to see several recommendations to make a contribution to set up future studies
  I will add some recommendations as suggested

# Peer review: Reviewer response for version 1

**DOI:** 10.5256/f1000research.28631.r71231

**Published:** 2020-09-21

**Authors:** Emanuele Angelucci

**Affiliations:** 1 Department of Oncology and Hematology, IRCCS Ospedale Policlinico San Martino, Genova, Italy

I have reviewed the document from my Egyptian colleagues with great interest. This paper is well written and addresses an important topic which is the clinical damage by iron overload and the efficacy of iron chelation therapy in a population of young children in the southern Mediterranean area.

 The article is clear and of relevance also because the population described is rarely seen in industrialized countries and consequently today rarely described in international medical journals.

 Furthermore, modern diagnostic technology is applied in a young hemoglobinopathy population suffering from severe iron-overloaded (probably inadequately chelated). Of relevance the conclusions are supported by an adequate (# 60) and well-studied age-matched control group.

 Although the main goal is, as expected, partially not achieved (correlation between pancreatic-hepatic iron with cardiac iron), there are several important conclusions from this article:

 Pancreatic iron concentration and liver iron concentration are good predictors of early cardiac function impairment.

 Very important: Cardiac function begins to deteriorate long before cardiac iron deposition is manifest by T2 * MRI even in a population of young children (mean age 13 years): this observation once again underlines the need for a regular (even intensive) iron chelation since initial stages of transfusion dependence.

 Correctly the authors use liver iron concentration as the standard for iron overload throughout the body ^1^.

 To improve paper readability, I suggest the following:

 Please clarify the definition of transfusion dependence (methods first paragraph).

 Few data are reported twice (methods and results; results and discussion). This is not necessary.

 The technical details of MRI and cardiac ultrasound are not needed. They can simply be referenced.

 Please be consistent with the definition of liver iron concentration (page 3: liver and pancreas T2 * versus second paragraph results). Liver iron concentration <2 mg / g dw is normal, liver iron concentration between 2 and 7 mg / g dw is mild overload.

 Figure 1 is unnecessary and can be deleted.

 Likewise, Tables 1 and 4 can be omitted.

 Table 5: missing caption.

 The discussion can be shortened.

 I don't understand reference no. 24.

 Include demographic details of the control population (age, gender, etc.).

 As a further question: Did the authors observe any difference between thalassemia and sickle cell disease?

Is the work clearly and accurately presented and does it cite the current literature?

Yes

If applicable, is the statistical analysis and its interpretation appropriate?

Yes

Are all the source data underlying the results available to ensure full reproducibility?

Yes

Is the study design appropriate and is the work technically sound?

Yes

Are the conclusions drawn adequately supported by the results?

Yes

Are sufficient details of methods and analysis provided to allow replication by others?

Yes

Reviewer Expertise:

Iron overload and iron toxicity. Hemopoietic cell transplantation.

I confirm that I have read this submission and believe that I have an appropriate level of expertise to confirm that it is of an acceptable scientific standard.

## References

[1] : Hepatic iron concentration and total body iron stores in thalassemia major. *N Engl J Med* .2000;343(5) : 10.1056/NEJM200008033430503 327-31 10.1056/NEJM200008033430503 10922422
